# Demonstrating Impact: Lessons Learned from the Queensland Aboriginal and Islander Health Council’s AOD-Our-Way Program

**DOI:** 10.3390/ijerph15030450

**Published:** 2018-03-05

**Authors:** Kimberly Cartwright, Dennis Gray, Eddie Fewings

**Affiliations:** 1 National Drug Research Institute, Curtin University, 7 Parker Place Technology Park, Bentley, WA 6102, Australia; d.gray@curtin.edu.au; 2 Queensland Aboriginal & Islander Health Council, 55 Russell St, South Brisbane, QLD 4101, Australia; eddie.fewings@qaihc.com.au

**Keywords:** evaluation methodology, Aboriginal community organisations, research capacity building, clicker technology

## Abstract

In this paper, we describe the innovative way in which the Queensland Aboriginal and Islander Health Council uses “clicker technology” to gather data to report on the key performance indicators of its “AOD-our-way” program, and how, with the subsequent combination of those data with other performance measures, it was possible to go beyond the initial evaluation. The paper also illustrates how the application of survey research methods could further enable enhanced reporting of program outcomes and impacts in an Indigenous context where Indigenous community controlled organisations want to build the evidence base for the issues they care about and ultimately drive their own research agendas.

## 1. Introduction

In this paper, we make the case for Indigenous and non-Indigenous organisations alike to integrate survey research methods into both the planning of activities and the evaluation of such activities using the Queensland Aboriginal and Islander Health Council’s (QAIHC) “AOD-our-way” Crystal Clear workshops as the case study. QAIHC employed “clicker technology”—a novel approach to gathering participant feedback before, during, and after each workshop in a pre- and post-test quasi-experimental design, and used the results to demonstrate that the key performance indicators in the funding agreement for the project had been met. The high demand for the Crystal Clear workshops in remote communities indicated both the critical need for methamphetamine resources in Queensland and the high value placed by workshop participants. The workshops were undoubtedly useful and relevant to participants, as noted by follow-up emails and written comments after the workshop, but evidence for whether the workshop resulted in *use of the materials*—the sole indicator of impact—is anecdotal rather than empirical because of the non-random selection of participants and the absence of individual identifiers of the participants in the before, during, and after “clicker” surveys. Hence, while QAIHC may have had an impact on participants’ knowledge about methamphetamine and their ability to use this knowledge to the benefit of the wider community, the methodological design of their self-evaluation meant that any change in knowledge, attitudes, and behaviours amongst the participants couldn’t be attributable to their exposure to the presentations in the workshop. Put simply, the Crystal Clear workshops may have had a positive impact on the building capacity among front line service workers to manage methamphetamine-related issues, but they did not have a *demonstrable impact*.

Clickers are hand-held wireless devices enabling individual participants in a lecture or similar setting to respond to questions posed by a presenter, and relay those responses to a computer. Invented in the 1950s for advertising and marketing purposes, audience response systems, including the clicker, resurfaced in the 2000s as an innovative learning tool in university auditoriums worldwide. While the clicker’s renaissance has been shown to increase student attendance and engagement [1,2,3,4,5] learning (as measured by both student self-reports and grades) [1,2,6,7,8], teacher efficiency [9,10], and pedagogical models (the “flipped classroom”) [10,11], its application is largely confined to the benefits they bring to both students and teachers, with little attention devoted to their methodological utility in furthering social science research [12]. To our knowledge, there is only one published work on the role of clickers as data collection tools for reporting purposes and the research ramifications of doing so [13].

When data collected from clickers in pre- and post-test research designs are used as key or supporting “evidence” for testing hypotheses or demonstrating outcomes, we argue that just as survey research principles guide the design and implementation of primary data collection tools, such as face-to-face surveys, they too should guide the application of clickers, especially in light of the emphasis placed on evidence in monitoring and reporting. Manipulating and analysing the data collected by the clickers, we show findings not revealed by the other methods QAIHC employed, such as participant observation and the reporting of data at face value (a common failing of many organisations that, for lack of funding, do not have their own dedicated research units). These findings illustrate the added value quantitative data analysis brings to evaluation, from the simple reporting of indicators to more sophisticated approaches, such as quasi-experimental research designs. They also hint at the possibilities for uncovering little known facts when scientifically valid and robust data are available for interpretation. Applying survey research principles and techniques at the start of a project or activity is particularly important if the data collected will be used for continuous learning processes and reporting. The value of building internal organisational capacity to conduct empirical research is great, and is particularly relevant for Indigenous community controlled organisations that want to build the evidence base for the issues they care about and ultimately drive their own research agendas. This is especially salient in Australia, where comprehensiveness of national datasets relating to Aboriginal and Torres Strait Islander People is often limited [14].

In May 2016, QAIHC and Queensland Health signed an agreement for QAIHC to undertake “Development and delivery of evidence-informed workforce training and education, clinical tools and electronic resources to support the implementation of frontline ‘ice’ (methamphetamine) initiatives in Queensland” [15]. For over a year, QAIHC and its partners developed tools and resources for alcohol and other drug (AOD) service workers and delivered two “dual diagnosis” workshops and 21 “Crystal Clear” workshops, across Queensland (increased from an original eight as a result of demand). Each workshop consisted of five presentations: What is “Ice”, Patterns and Prevalence of “Ice”, Effects of “Ice”, Interventions, and Resources.

QAIHC conducted its own evaluation of the Crystal Clear workshops based on clicker technology to assess the mastery of the information presented and a follow-up survey using Survey Monkey (SM) to ascertain subsequent use of the electronic resources provided. These data were used to demonstrate to Queensland Health that program key performance indicators (KPIs) had been met. QAIHC asked the National Drug Research Institute (NDRI) to conduct a more formal evaluation to document lessons learned and to make recommendations for improving the design and delivery of future workshops. The NDRI evaluation was based on QAIHC’s own evaluation materials, focus groups with workshop participants in four locations, and interviews with the QAIHC program manager and the two workshop presenters.

QAIHC used participant self-reports ascertained by the clickers “before, during, and after” the five presentations comprising each workshop as ‘evidence of knowledge transfer’ for a number of outcome measures [16]. For example, the first presentation, “What is Ice?” focused on the pharmacological properties of methamphetamine and distinguished between crystal methamphetamine and other forms of the drug. Prior to listening to the presentation, participants were asked to rate themselves on a scale of 0 (no knowledge) to 10 (excellent knowledge) regarding their knowledge about ice (the “before” survey). Immediately after the presentation, the same participants were asked to rate themselves regarding their knowledge of ice (the “during” survey). They were then asked to rate themselves at the end of the workshop (the “after” survey). Based on the responses, QAIHC was able to show that there was an 83% increase in the number of participants demonstrating satisfactory levels of knowledge (those with a score of 7 and higher). Similarly impressive results were reported for the other measures.

QAIHC also used a follow-up online questionnaire in Survey Monkey (SM) to collect data about participants’ use of the informational resources provided to them on a USB flash drive. After each workshop, facilitators informed participants that they would receive the web link to the questionnaire in six to ten weeks following the workshop and were asked to report on how they were using the resources. Forty-three percent of participants completed the follow-up survey, of whom 82 percent (*n* = 107) reported using the resources when working with clients and found them to be helpful and positive, resulting in an impact estimate of 35%. Subsequent use of the workshop materials in the workplace was the only measure of “impact” listed among the KPIs in the funding agreement [15].

In this paper, we discuss two areas in which QAIHC’s methodological approach could be improved (Table 1). The first area concerns the need for a random sample of workshop participants. While we acknowledge that the pool of participants attending a one-day workshop is not generally subject to the principle of random selection, it was essential in this instance because QAIHC employed a pre- and post-test quasi-experimental evaluation design, a methodology that requires a random sample of participants with similar motivation levels. Furthermore, the funding agreement explicitly stated that the workshops would upskill individuals representative of AOD and related frontline workers in Queensland. That the workshop participants may not be representative of the AOD front-line population in Queensland is important because the needs, concerns, and capabilities of the participants as expressed at the workshop may not reflect those of the wider AOD workforce, potentially resulting in the identification of priorities and initiatives that are based on the views of some sections of the workforce at the expense of others. Another problem is the uneven distribution of knowledge transfer and its subsequent use on the ground. If, for example, there is an overrepresentation of police and an underrepresentation of psychologists, not only may the materials be less relevant for the participants, but their use may be concentrated in the criminal justice sector at the expense of the AOD sector. Hence, the non-random recruitment of Crystal Clear workshop participants has implications on both the assumptions and conclusions that can be made. 

The other shortcoming was the non-collection of socio-demographic data about the workshop participants, which combined with the absence of individual identifiers, meant that it was not possible to identify and link individual responses. The data analysis was therefore limited to site-level analyses, in which the distribution of responses to questions could be compared to see if there were any statistically significant bi-variate correlations. Having only site-level data available is problematic because the potential for making spurious conclusions is high. Aggregate data can mask relationships and underlying behaviours that are not directly observable, an issue addressed in the Discussion. 

In the face of these limitations, we approached the evaluation by asking three key questions in an attempt to uncover as many of the findings as was possible with site-level data. We could have concluded that any findings were erroneous due to the non-random nature of the sample and stopped the evaluation altogether. We chose, however, to forge on with the analysis, with the caveat that the data may not be representative of the service worker workforce in Queensland, in order to illustrate the value that quantitative analysis has in revealing knowledge about attitudes and behaviours that often go unnoticed because they are not readily apparent. 

Our first question was whether knowledge transfer took place in the workshops. The report on the project, submitted by QAIHC to and accepted by Queensland Health, provided sufficiently compelling evidence to conclude that participants had gained new knowledge about crystal methamphetamine as a result of attending the workshops. This was later confirmed in the focus group interviews conducted by NDRI with participants at several of the sites.

Our second question concerned the extent of the impact that could be demonstrated by participation in the Crystal Clear workshops and the subsequent use of the workshop materials by the participants in their daily work with methamphetamine users and their families. The moderately low response rate to the SM questionnaire in which just 35 percent of respondents reported that they were using the workshop materials in their daily work posed a dilemma. Had 70 percent of participants completed the SM questionnaire and reported they were using the informational resources in their work and found the materials to be useful, it would have been reasonable to conclude that the workshops had a high impact value. However, because less than 50 percent of participants completed the questionnaire, and even fewer reported using the materials, issues of selection bias and nonresponse became central to our evaluation. For instance, could it be assumed that the participants who completed the questionnaire were representative of the larger group of participants who did not? If the data analysis revealed an affirmative response, then the case could be made, albeit a tenuous one, that the impact of the workshops exceeded the 35% estimate and may be as high as that reported in the responses to the SM questionnaire (82%).

Our third question also related to program impact. The intention behind the workshops was to equip a cross-section of front-line service workers to meet the needs of methamphetamine-using clients and their families. Was workshop attendance sufficiently high and representative of the AOD and related front line workforce in Queensland for participants to make a difference on the ground? If the answer is “no”, it begs the question why the workshops had been conducted at all. Unfortunately, there is no way of knowing the answer to this question without commencing with a new round of workshops using principles of random selection to ensure statistical representativeness, a solution that is both costly and impractical. Because one cannot simply “fix” purposive samples and make them representative, questions about statistical representativeness in the context of evaluation are pivotal and is precisely why survey research principles should guide program and evaluation design from the start.

Within this conceptual framework, we queried, “What is possible to say about program impact?” Ultimately, funders want to know what the program budget financed and what was accomplished. They want evidence to justify the use of the expenditure in light of competing alternatives and are hence increasingly calling for numeric estimates of impact. We focused our attention on the second question regarding nonresponse and developed hypotheses about possible selection biases among the group that completed the SM questionnaire. For example, were there any reasons why the majority of participants did not complete the questionnaire—reasons setting them apart from participants who did? These reasons include the passage of time and the window of exposure participants had to utilise the informational materials; variances in reported participant satisfaction levels during the time trajectory of the workshops; and participants’ understanding of the workshop presentations and their motivation to complete the follow-up SM questionnaire.

If there were no differences between the group of participants who had completed the SM questionnaire and the group who had not, then the case could be made that the rate of use of the USB materials was possibly higher than the 35% estimate provided by the SM survey responses. For example, one could make the assumption that because there were no differences between the two groups, the rate of use as reported in the follow-up survey (82%) or a fraction thereof could be applied to all participants. 

## 2. Materials and Methods 

Three primary data sources informed this case study, all of which presented limitations to the evaluation conducted by NDRI. The first, based on the results of the clicker technology, was an Excel spreadsheet containing the participant responses to the ‘before, during, and after’ questions asked to participants in the workshops. These data were aggregated into variables differentiated by workshop location. For example, participants used the clicker to rate how well they understood each of the five presentations on a scale of 1 to 10, where 1 is “not at all” and 10 is “completely understood”. QAIHC pooled the responses at each site to construct a summary variable on comprehension, ‘I was able to understand the content covered (all sections combined)’. This variable contained the distribution of responses from 1 to 10 for all the presentations at each site. It did not tell us, however, if multiple responses with a particular rating to a question came from one or different individuals, nor did we know whether a particular individual also gave the same or similar ratings to other questions. 

The second data source was the responses to the follow-up SM questionnaire. In the absence of individual identifiers, it was not possible to link the data collected from the clickers about participants’ views of the Crystal Clear workshop presentations with responses to the follow-up SM questionnaire. The information from the SM questionnaire revealed that the majority of participants who were using the workshop materials in their daily work found them to be helpful; however, we do not know how these respondents viewed the Crystal Clear workshop presentations as reported by the clickers. 

The third data source was a record of registered participants—by name, their place of work, and job title—attending each workshop site. Although information on Aboriginality was not collected, the majority of the participants were Aboriginal. The participant record provided very little useful information because it could not be linked to individual responses. Furthermore, from our interviews with QAIHC, we learnt that each participant was recruited by the Chief Executive Officer of his/her employing service organisation. There were no formal selection criteria and the methods used to recruit the employee-participants varied, and are largely unknown. For example, some CEOs required all of their staff to attend the workshop in their local area, while others selected employees on the basis of their job roles, availability, participation in previous trainings, outcome of a mental health risk assessment, and other criteria.

Not knowing the circumstances in which participants were selected to attend the workshops precludes knowledge about the motivational levels of participants to attend the workshop, an important control variable in pre- and post-test evaluation design because motivation is linked to engagement and information take-up. Most importantly, due to the uncontrolled recruitment of participants, it is unknown whether they represent AOD and related frontline workers in Queensland.

In contrast to the self-evaluation in which the clicker data were simply reported as aggregated descriptive information, we utilised the data to shed light on the factors that might have influenced site-specific response rates to the SM questionnaire. From the clicker data, we constructed variables based on participant responses and workshop attributes, such as the date of the workshop, the number of participants at each site, and self-reports on participants’ understanding of the materials and their confidence to engage with clients about methamphetamine using the materials as aids. The construction of the most important of these variables is described as follows and presented in Table 2. 

An event time variable was constructed by counting the date of the first workshop as “1” and cumulatively adding the number of days between the workshops. This method takes into account the length of time that transpired between workshops and gives an accurate record of the amount of “exposure” time participants had to complete the follow-up SM questionnaire. The only restriction from completing the online questionnaire was the six to ten week waiting period after the workshops. Thus, unless they completed it immediately, participants from earlier workshop dates had longer windows of opportunity to complete the questionnaire than those with later workshop dates.

A dummy variable was constructed to distinguish those sites whose participants rated their understanding of one or more workshop presentations to be low (a score of 1 to 4) from those sites whose participants gave ratings of 5 and above (“Don’t understand”). The dummy variable was coded “1” if the site had participants who gave any low scores and “0” if otherwise. To capture participants’ views about the workshop and about their confidence in using the materials, for each site, we counted the number of responses with scores of 7 and above (seven was the minimum value QAIHC used to define competency) and divided them by the number of participants. These variables are expressed in proportional units to account for differences in the number of participants across sites. For example, the variable corresponding to the question asking participants to rate the facilitators’ knowledge has been redefined and coded as “the proportion of all participants at the site assigning the facilitator a score of 7 to 10”. The response rate variable was coded at the site level, and was calculated as the proportion of workshop participants at each site that completed the SM follow-up questionnaire. 

Finally, the data were weighted according to the proportional representation of the participants at each site (the combined number of participant responses at each site ranging from 32 in Gladstone and 157 in Innisfail) divided by the total number of combined responses across all the sites (*n* = 1733). Accordingly, the sum of the weights equals 1. We chose basic bivariate correlations (with two-tailed significance) and *p*-values to test the strength of the associations, if any, between two variables to lend support for our hypotheses. The data were analysed using SPSS (Armonk, NY, USA).

## 3. Results

The findings indicate that, based on the clicker technology, participants had qualitatively different experiences at the workshops, which influenced whether or not they responded to the SM questionnaire (Table 3). Participants at the earlier workshops were more likely to report lower satisfaction levels with the workshops, give lower ratings to the presenters, and report poorer understanding of the workshop materials compared to participants who attended the later workshops. Despite this, participants at the earlier workshops were more likely to complete the SM questionnaire. This may be explained by the fact that these participants had more time to use the workshop materials with clients compared to participants who attended the later workshops. Furthermore, the impetus for the ‘AOD-our-way’ workshops originally grew out of demand by QAIHC member organisations that were sites of the ‘early’ workshops. Having longer exposure to the ideas behind the presentations, participants may have felt more ownership over the informational resources than participants from the other sites. The participants attending the earlier workshops also scored themselves as being more confident to manage ice issues and to access ice resources than the participants attending the later workshops. 

A more detailed analysis of clicker responses to participant understanding of the content of specific presentations is consistent with the findings reported in Table 3. Of the five presentations, the most difficult for participants to understand were the first two, “What is Ice?” and “Patterns and Prevalence of Ice”. Since a higher percentage of participants in the later workshops understood these presentations, it is not surprising that they reported higher confidence in their knowledge about ice than participants in the earlier workshops.

Satisfaction levels and engagement with the content, as measured by the clicker responses, were also important factors driving completion of the SM questionnaire. As expected, participants who reported higher overall satisfaction scores with the workshops were more likely to participate in the follow-up survey than participants who gave lower satisfaction scores. Equally important, participants were less likely to participate in the survey if they had a limited understanding and appreciation of the content presented. Participants in the later workshops scored the presenters’ knowledge of the material to be higher than participants in the early workshops, and they reported feeling more engaged than participants in the early workshops.

Results derived from the clicker technology enabled us make the following two inferences. First, participants who reported low comprehension of one or more presentations were less likely to use the informational resources. Second, participants who used the resources in their work following the workshop were more likely to complete the SM questionnaire than those who had not. These results suggest that attributes of self-selection may partly underlie site level response rates to the questionnaire. That is, the group of participants who completed the questionnaire may be self-selected for characteristics that set them apart from the larger group of participants who did not complete the questionnaire. Hence, it would be imprudent to assume that all the workshop participants used the resources and perceived them in the same positive light as the SM respondents. To summarise, the findings indicate that the two groups are statistically different from each other; hence, on this basis we cannot generalise from the group who used the materials to all participants in the workshop. There is only evidence pointing to an impact estimate of 35 percent, as indicated by the responses to the SM questionnaire.

## 4. Discussion

This case study has shown that valuable information about workshop processes can be ascertained from rudimentary site level data. From our interviews with the two workshop presenters, we know that they reflected after each workshop with the intent of improving the next. They appeared to learn over time and the quality of the workshops towards the end of the program was likely to be an improvement over the first two or three. These findings are consistent with participant feedback from the clickers. Although the presenters spent dedicated time for reflection, they did not anticipate the findings from the NDRI evaluation regarding program impact. This may be because they were so intimately involved in the running of the workshops over the ten-month period. Due to the expressed need for “ice” resources among Aboriginal community organisations in the outer regions, which led QAIHC to expand the scope from 8 to 21, the presenters believed that they impacted many more participants than the estimated one-third. They were therefore astonished to learn from the focus group interviews we conducted that many of the participants had put their USB flash drives in a drawer and forgotten about them. This alone points to the value of conducting evidence-based social science research:

*We can’t solve our social problems until we understand how they come about and persist. Social science research offers a way of examining and understanding the operation of human social affairs. It provides points of view and technical procedures that uncover things that would otherwise escape our awareness. Often, as the cliché goes, things are not what they seem, and social science research can make that clear [17]*.

Response rates to the follow-up SM questionnaire were higher among participants in the earlier workshops despite their relatively lower satisfaction levels, suggesting that workshop fatigue amongst the participants may have set in by the time the later workshops were offered. A number of participants interviewed could not recall the details of the workshops and had to be reminded by their colleagues. It was made clear that the participants had attended numerous workshops and trainings about methamphetamine and related issues in the calendar year and some could not distinguish them. This suggests there may have been a saturation point at which participants could no longer absorb new lessons or retain what they had learnt. Interviews with participants indicate that greater coordination of workforce development trainings across organisations working in the same sector and within organisations to ensure the appropriate mix of trainings per staff hour is required for sufficient uptake by participants to apply the lessons as intended. In addition, there is a need for follow-up learning opportunities for participants to retain the key learnings from the workshop and put them into practice and for the organisations participating in the workshops to incorporate the learnings into their case management models.

While the data analysis has offered valuable lessons about how the conduct of the workshops might have influenced participants’ use of the information in their work (program impact), much more information could have been ascertained with fewer assumptions had the workshop design incorporated survey research principles at the start. The first lesson for organisations is to investigate the requirements of the evaluation methodology and choose a methodology that is fit for purpose, practical, and feasible. For example, an alternative to the pre- and post-test quasi-experimental design QAIHC used is a short questionnaire on knowledge, attitudes, and behaviours delivered to each participant before and after the workshop via clickers or paper questionnaires. 

The second lesson is to collect information about individual participants (e.g., gender, age, organisational type of employer, Aboriginality, etc.) and analyse the data for co-variates and potentially causal relationships. This would enable workshop organisers to see if they need to adjust the presentations to specific participant needs and to understand the differential impacts the presentations had on participant groups. Had QAIHC collected information about each participant, it would have been possible to link the clicker responses to the workshop with those to the follow-up SM questionnaire. This would have facilitated a much richer analysis of the data, potentially leading to findings that could be explored further in the quest to improve organisational responses to methamphetamine use and its harms.

While QAIHC can be commended for using the clicker technology, which has several important advantages over paper surveys, such as its automated collection of responses in electronic data form, therefore obviating the need for manual data entry of questionnaires, QAIHC did not use the clickers to their full potential. For example, clicker data collected in the first set of workshops could have been analysed early on to identify any issues in need of rectification, such as a low understanding of particularly difficult concepts, what these were, and their positioning within the workshop program. The presenters could also have used the feedback from the clickers to tailor their instruction in real-time, such as slowing down, encouraging participants to ask questions within the wider group setting or in private. The clickers could also have been used at the start of the workshop to obtain participant characteristics using the technology to link the individual clicker number with the participant’s socio-demographic data, thus bypassing the need for participants to disclose their names. 

QAIHC presenters also encountered several disadvantages using the clicker technology, including user error, problems with reception in remote areas, and unexplained variations in response rates. For example, some of the participants had difficulty pressing the intended buttons on the clicker and interrupted the session to alert the facilitator that they meant to give a rating of 10 but the clicker only read the 0. This fortunately served as an early warning signal to QAIHC of the necessity to alert participants to make sure the clicker read the intended response. In one instance, the clicker technology did not work in a remote area experiencing inclement weather conditions. We discovered in our analysis that the clicker may not have captured the data from all of the participants providing feedback as part of the final end of session survey. Although the facilitators stated that the vast majority of participants completed the workshop in its entirety, the response rate across all 21 sites to the post workshop survey was only 83 percent, with a range of 48 percent at Thursday Island to 100 percent at Gladstone and Toowoomba. One can conclude that pre-testing the clicker technology in different environments is important, as well as having paper questionnaires on hand in the event of system failure.

## 5. Conclusions

This case study has shown that the choice of an evaluation methodology has implications for the design of project, workshops, and other activities. Before committing to an evaluation methodology, it is important to investigate the requirements of the approach against organisational capabilities to implement it to a high standard. In the QAIHC example, clickers were used as data collection tools to report against KPIs using a quasi-experimental pre- and post-test design. The error in implementation was not due to the employment of the clickers, but in a failure to meet the requirements of the quasi-experimental design. Even the simplest evaluations are based on survey research principles, and for this reason alone, organisations, intent on conducting their own evaluations, should consider hiring researchers skilled in evaluation methodologies and/or building the capabilities of existing staff in this area.

There are trade-offs to consider given the effort required to apply social science methods to study designs and these costs must be balanced against the benefits. Knowing the costs of not applying a certain technique, such as the random recruitment of participants, is also important; and in some situations, econometric methods can be used to adjust data so that they are representative of the true population, as long as a minimum amount of data are collected. Econometrics cannot remedy the inherent failing of a purposive sample, however, which is why it is critical that issues of representativeness are addressed as much as practically possible in the design of evaluations, especially when the results are used to demonstrate outcomes and impact.

This case study has shown that it is possible for Indigenous organisations to collect personal data from their participants using culturally safe approaches which are also pragmatic and fit for purpose. The benefits of having individually linked data are great. Crucially, they allow for causal connections to be made between phenomena important for the testing of ideas beyond the classroom environment to policy making in practice. 

While the novel application of the clicker technology to performance reporting by QAIHC was the impetus for this case study, it touches on issues extending beyond the parameters of good practice evaluation design—issues such as the centrality of research evidence as a driver of change and the need for Indigenous community organisations to build their research capabilities in-house so that they can start collecting empirical data which can be used to tell their stories in their own way.

## Figures and Tables

**Table 1:** Methodological shortcomings in the pre- and post-test design and consequences for the self-evaluation results.

Shortcoming	Consequence
Non-random sample of participants	It is unknown whether the participants represent AOD front-line workers in Queensland. If they are not representative, then the employ of the workshop materials may be non-random and concentrated in one sector leading to non-efficacious results. Mandatory recruitment also raises the question as to the motivational level of participants, especially those whose duties do not involve significant client engagement (e.g., receptionists).
Non-collection of participant’s socio-demographic information and absence of individual identifiers	Severely limits data analysis and heightens the possibility for erroneous conclusions to be made. Precludes any possibility of linking responses.

**Table 2:** Description of variables.

Variable Name	Description
Event time	Interval variable that marks the date of the workshop
Low understanding	Low understanding of one or more presentations. Dummy variable (1 = low understanding, 0 = other)
Confident in knowledge of ice	Proportion of participants at each site who gave themselves a score of 7 and higher on their knowledge of ice
Confident to manage ice issues	Proportion of participants at each site assigning a 7 and higher rating in their own confidence to manage ice issues in their workplace
Confident in ability to access ice resources	Proportion of participants at each site assigning a 7 and higher rating in their own confidence to access ice resources
Found presenters to be “knowledgeable”	Proportion of participants at each site who rated the presenters to be knowledgeable with a score of 7 and higher
Found workshop “engaging”	Proportion of participants at each site who rated the workshop as a whole to be engaging with a score of 7 and higher
Response rate	Proportion of participants at each site who completed the follow-up SM questionnaire

**Table 3:** Summary statistics of results using clicker technology at the “AOD-our-way” workshops.

Correlations	*p*-Value (sig.)
Event time & response rate to SM questionnaire	−0.176 (0.01)
Event time & overall satisfaction	0.333 (0.01)
Overall satisfaction & response rate	0.288 (0.01)
Low understanding & response rate	−0.357 (0.01)
Low understanding & event time	−0.114 (0.01)
Confident in ability to manage ice issues & event time	−0.164 (0.01)
Confident in knowledge of ice & event time	0.323 (0.01)
Confident in ability to access ice resources & event time	−0.448 (0.01)
Found facilitators “knowledgeable” & event time	0.420 (0.01)
Found workshop “engaging” & event time	0.297 (0.01)

## Abbreviations

The following abbreviations are used in this manuscript:

QAIHC	Queensland Aboriginal and Islander Health Council
NDRI	National Drug Research Institute
CEO	Chief Executive Officer
KPIs	Key Performance Indicators
SM	Survey Monkey
AOD	Alcohol and Other Drugs
SPSS	Statistical Package for the Social Sciences
